# Report of *Giardia duodenalis* in a non-captive chital *Axis axis* (Erxleben 1777) in Santa Catarina, South of Brazil

**DOI:** 10.29374/2527-2179.bjvm003224

**Published:** 2024-09-11

**Authors:** Jackson Fábio Preuss, Clóvis Junior Chimin Chafes, Andréia Buzatti

**Affiliations:** 1 Biologist, DSc., Núcleo de Estudos em Vida Selvagem (NEVS), Universidade do Oeste de Santa Catarina (UNOESC). São Miguel do Oeste, SC. Brazil.; 2 Veterinarian, MSc, Resident. Programa de Residência Multiprofissional – Clínica Médica, Cirúrgica e Anestesiologia de Animais de Companhia, Universidade Federal de Jataí (UFJ). Jataí, GO. Brazil.; 3 Veterinarian, DSc., Curso de Medicina Veterinária, UNOESC. São Miguel do Oeste, SC. Brazil.

**Keywords:** giardiasis, zoonotic agent, public health, parasitological diagnosis, exotic deer, giardíase, agente zoonótico, saúde pública, diagnóstico parasitológico, cervo exótico

## Abstract

Giardiasis is an infection of the small intestine by protozoa of the genus *Giardia*, which has a wide range of susceptible hosts, including domestic and wild animals and humans. *Giardia* is a zoonotic agent and represents one of the main human parasites, with high prevalence and great importance in public health. This report aims to describe the parasitism of a non-captive Chital deer (*Axis axis*) by *Giardia duodenalis.* The animal, after being rescued by soldiers from the 2nd Platoon of the 2nd Company of the 2nd Battalion of the Environmental Military Police of the State of Santa Catarina, was sent to the Wildlife Studies Center (NEVS) of the University of Western Santa Catarina (UNOESC). During clinical care, an exposed fracture in the left pelvic limb and signs of acute respiratory failure were found. Fecal samples were collected for later parasitological diagnosis. Two techniques were applied: centrifugal flotation with zinc sulfate, to diagnose parasites of the gastrointestinal system, and Baermann, to search for parasitism in the respiratory tract. The investigation revealed the presence of *Giardia duodenalis.* The animal died on the same day of its arrival due to a cardiorespiratory arrest. The presence of this parasite in an invasive exotic deer species highlights its epidemiological importance, as it can act as a source of infection and spread the disease to humans and other animals.

## Resumo

Giardíase é uma infecção do intestino delgado por protozoários do gênero *Giardia*, o qual apresenta uma ampla variedade de hospedeiros suscetíveis, incluindo animais domésticos, selvagens e também humanos. *Giardia* é um agente zoonótico, e representa um dos principais parasitas de humanos, com alta prevalência e grande importância em saúde pública. Este relato tem como objetivo descrever o parasitismo de um indivíduo de cervo Chital (*Axis axis*) de vida livre por *Giardia duodenalis.* O animal, após ser resgatado por soldados do 2º Pelotão da 2ª Companhia do 2º Batalhão da Polícia Militar Ambiental do Estado de Santa Catarina, foi encaminhado ao Núcleo de Estudos em Vida Selvagem (NEVS) da Universidade do Oeste de Santa Catarina (UNOESC). Durante o atendimento clínico foi constatada fratura exposta no membro pélvico esquerdo e sinais de insuficiência respiratória aguda. Amostras de fezes foram coletadas para posterior diagnóstico parasitológico. Para isso, foram usadas duas técnicas: centrífugo-flutuação em sulfato de zinco, com o objetivo de diagnosticar parasitas do sistema gastrointestinal, e Baermann, com o objetivo de pesquisar parasitas do trato respiratório. Os achados revelaram presença de cistos de *Giardia duodenalis.* O animal morreu no mesmo dia de sua chegada, devido a uma parada cardiorrespiratória. A presença desse parasito em uma espécie de cervídeo exótico invasor destaca sua importância epidemiológica, pois o mesmo pode atuar como fonte de infecção e de disseminação da doença para o ser humano e/ou outros animais.

## Introduction

*Axis axis* (Erxleben, 1777), also known as Chital, is a deer species native to Asia (India, Nepal, and Sri Lanka). It is medium in size, with males reaching a shoulder height of 80-100 cm and a length (excluding the tail) of 119-185 cm, while females are 67-87 cm tall and 114-147 cm long. Adults have a reddish-brown coat with white spots (Nowak, 1999; Schaller, 1967).

Deer, in general, are among the most intentionally introduced vertebrates on the planet (Clout & Russell, 2008). The Chital, in particular, was introduced in several countries as an ornamental species, as a food resource, and for sport hunting (Mares & Ojeda, 1984), given its much-appreciated meat and its horns serving as trophies (Belden, 1994). It has been successfully introduced in Europe, Australia, Java, New Guinea, Mexico, Argentina, Uruguay, the United States, and the Andaman Islands (Achaval et al., 2007; Bentley & Downes, 1968; De Vos et al., 1956; Faas & Weckerly, 2010; Grubb, 2005; Long, 2003; Novillo & Ojeda, 2008; Romero et al., 2008). In Brazil, its presence was recorded for the first time in 2009 in areas of the Pampas, in the extreme west of the state of Rio Grande do Sul (Sponchiado et al., 2011), and in 2020 in the Atlantic Forest of the extreme west of the state of Santa Catarina (Preuss et al., 2020).

Chital occurrence potentially threatens the ecosystem and local species (Preuss et al., 2020; Sponchiado et al., 2011). Studies have indicated potential negative impacts on various plant and animal species (Davis et al., 2016). The most significant impacts likely occur through interspecific competition for habitats and food resources with other herbivores, whether domesticated or wild (Bartoš et al., 1996; Stephens et al., 2003). Thus, they can also play a significant role in the transmission of parasites to these animals, especially of those with oro-fecal transmission (Bengis et al., 2002; Cripps et al., 2019; Davis et al., 2016; Faas & Weckerly, 2010; Mohanty et al., 2016; Smith et al., 2009).

Among parasitic diseases, we can mention Giardiasis, an infection in the small intestine caused by protozoa *G. duodenalis* (syn. *G. intestinalis*, *G. lamblia*) (Seabolt et al., 2022), which parasitize a wide variety of hosts amid domestic and wild animal species, from mammals to birds and amphibians (Santin, 2020), with more than 40 animal species susceptible to becoming hosts (Horlock-Roberts et al., 2017; Ryan & Cacciò, 2013). In addition to having a wide range of hosts, *Giardia* is considered one of the main human parasites, with high prevalence and great importance in public health (Adhikari et al., 2021). According to Einarsson et al. (2016), in humans, giardiasis is responsible for about 280 million cases of diarrhea per year worldwide.

To date, *G. duodenalis* is phylogenetically classified into eight distinct assemblages (A to H), or genotypes, where A and B are infectious to humans and animals, while assemblages C–H show specificity for specific animal hosts: C and D are specific to dogs and other canids, E is found in ungulates including cattle, F is found in cats, G is found in rodents, and H is found in marine mammals such as pinnipeds (Cacciò et al., 2018). However, this is a dynamic scenario, as recently assemblages C, D, E, F, and G have also been found in human infections (Buret et al., 2020; Chang et al., 2023; Fantinatti et al., 2016; Garcia-R et al., 2021; Higuera et al., 2020), suggesting that *G. duodenalis* assemblages may have a broader zoonotic potential than initially assumed (Reis et al., 2023). However, the genotypes are morphologically indistinguishable and can only be differentiated by genetic analysis (Cacciò & Ryan, 2008).

This study aimed to report the presence of *G. duodenalis* in a non-captive Chital deer, which may threaten the health of other animals and humans.

## Case report

**Figure 1:**
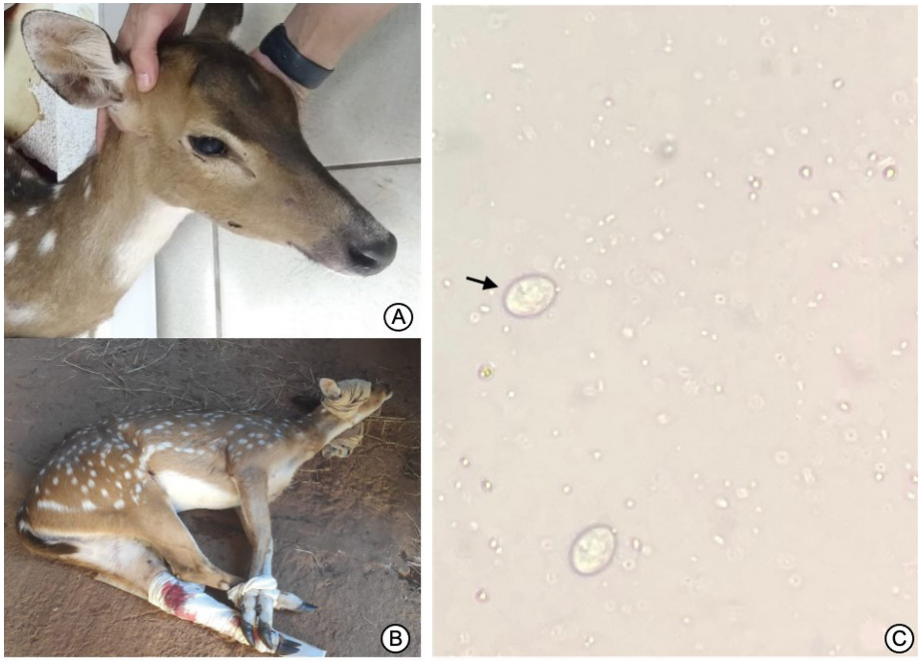
(A) and (B) Specimen of *Axis axis* (Erxleben, 1777) rescued by 2nd Platoon of the 2nd Company of 2nd Battalion of the Environmental Military Police of the State of Santa Catarina; (C) *Giardia duodenalis* cysts. Source: Personal archive.

On August 30, 2019, a female Chital (Figure 1A and 1B) was taken to the Wildlife Studies Center (NEVS) at the University of Western Santa Catarina (UNOESC). It had been rescued by soldiers from the 2nd Platoon of the 2nd Company of the 2nd Battalion of the Environmental Military Police of the State of Santa Catarina after being found in a rural area near the BR-163 highway in the municipality of São José do Cedro (26°28’S, 053°30’W)), state of Santa Catarina, Southern Brazil.

For inspection and physical examination, the individual was sedated using dex-medetomidine 0.01mg/kg as a pre-anesthetic medication followed by Ketamine 7mg/kg + Midazolam 0.3mg/kg, all used intramuscularly in a single application for dissociative anesthesia. The physical examination revealed that the animal had an exposed fracture on the left pelvic limb and signs of acute respiratory failure. Feces were collected rectally, stored in procedure gloves, identified, and refrigerated until the moment of analysis, carried out the following day. Macroscopic examination of the feces demonstrated normal consistency, color, and odor. The samples were processed by two techniques using fresh feces: centrifugal flotation with zinc sulfate (Faust et al., 1938), which was used to diagnose parasites of the gastrointestinal system, including protozoa (Monteiro, 2017), and Baermann (1917), to search for parasitism in the respiratory tract. The first technique revealed the presence of cysts of *G. duodenalis* (Figure 1C), while the second revealed the absence of parasitic structures. The cysts were identified using a binocular microscope with a 40x objective. Their identification was based on literature records (Taylor et al., 2017). The animal died on the same day of its arrival due to a cardiorespiratory arrest.

## Results and discussion

The results of this study provide the first descriptions of the occurrence of cysts of *G. duodenalis* on a free-living chital deer.

After an initial coproparasitological examination of the specimen, we observed the presence of cysts of *Giardia*, a flagellate protozoan that presents two developmental stages, trophozoites and cysts. Trophozoites represent the active and mobile form of the protozoan and are found in the small intestine of hosts. Cysts derive from the encystment of trophozoites, are eliminated in the feces of the parasitized host, and represent the dormant form of the protozoan that resists in the environment (Adam, 2021; Monteiro, 2017).

The main clinical signs that animals parasitized by *Giardia* may present include diarrhea, which is usually intermittent, and impaired digestion and absorption of nutrients, which may lead to dehydration, weight loss, and even death (Bowman, 2010). The animal in the present report did not show clinical signs compatible with giardiasis, which is in line with Certad et al. (2017). According to the author, although this parasite has a high prevalence, not all infected individuals show clinical signs. Several host-associated factors influence clinical manifestations, such as nutrition and body condition score, age, immunological response, co-infections with other gastrointestinal pathogens, and microbiota composition. However, according to Monteiro (2017), even asymptomatic individuals can eliminate infectious forms via feces and thus promote the spread of the disease. That is, they can act as a source of infection.

A non-captive animal, such as the one referred to in the present report, parasitized by an agent with several host species and zoonotic potential, has great epidemiological importance because it can act as a source of infection and dissemination of the disease. According to Coffey et al. (2021) and Einarsson et al. (2016), the development cycle of the parasite is direct, which facilitates its propagation, and the infection of susceptible hosts occurs through ingestion of water or food contaminated by cysts of the agent, which survive for several months in the environment.

Currently, the role of wild animals in the epidemiology of giardiasis is little known (Lallo et al., 2009). However, molecular studies indicate that some of these animals may represent sources of infection for other animals and humans (Sulaiman et al., 2003). Although the occurrence of *Giardia* sp. in free-living chital has not been previously reported, there are reports of the parasite infecting captive chital deer and other deer species. For example, Karim et al., (2021), detected the occurrence of *G. duodenalis* in chital deer kept in captivity in Bangladesh. Lalle et al. (2007) reported the prevalence of this protozoan in fallow deer (*Dama dama*). García-Presedo et al. (2013) reported positive samples for *G. duodenalis* in roe deer (*Capreolus capreolus*). In the state of São Paulo, Holsback et al. (2013) reported *Giardia* in a non-captive gray-brocket deer (*Mazama gouazoubira*). In Norway, red deer (*Cervus elaphus*) were infected by *G. duodenalis* (Hamnes et al., 2006). In the United States, a white-tailed deer (*Odocoileus virginianus*) tested positive for *G. duodenalis* (Santin & Fayer, 2015). Song et al. (2018) reported the prevalence of *G. duodenalis* in dwarf musk deer (*Moschus berezovskii*) in China.

Like their hosts, parasites can occasionally expand their geographical area abruptly in an invasion process, thus promoting widespread contamination of the environment (Chalkowski et al., 2018; Hatcher et al., 2012; Poulin, 2017). The occurrence of *Giardia* sp. in Chital, an invasive exotic animal, can promote environmental contamination and lead to the infection of domestic animals. In addition, it is also worrying for public health since giardiasis is a zoonosis. Because many parasites that affect humans are directly or indirectly transmitted by animals, knowing the epidemiology of parasitic zoonoses, emphasizing here giardiasis, is essential for public health.

The chital deer is considered the most successful generalist invasive animal among deer species, with successful introductions documented in several parts of the world (Etges et al., 2023). It is considered a predominantly generalist wild deer, easily habituated to human presence, and herds often gather in open areas, invading urban and peri-urban environments (Duckworth et al., 2015; Moriarty, 2004). These deer represent a concern for both economic and environmental reasons due to possible competition with cattle and as a disease vector (Cripps et al., 2019; Davis et al., 2016; Debárbora et al., 2012; Huaman et al., 2023; Mertins et al., 2011; Richardson & Demarais 1992; Riemann et al., 1979), as well as due to interspecific competition with native fauna (Ali, 2004; Faas & Weckerly, 2010; Mohanty et al., 2016).

Environmental changes, represented here by the introduction of exotic species, can compromise ecological niches and create conditions that allow the spread of diseases, including parasitic zoonoses (Cripps et al., 2019; Patz et al., 2000). Understanding this knowledge gap is essential to establish appropriate management strategies for deer, minimizing possible impacts of the species on human health (Huaman et al., 2021).

In this sense, identifying pathogenic agents in exotic animals with invasive potential is fundamental to understanding their epidemiology and adopting preventive measures.

## Conclusions

This study demonstrated the occurrence of *G. duodenalis* in a non-captive chital deer in Santa Catarina, southern Brazil. To our knowledge, this is the first report of infections in non-captive *Axis axis*. This demonstrates that the host range of this parasite is broader than previously reported. The presence of this parasite in an invasive exotic deer species indicates a potential for transmission of *Giardia* to humans and/or other animals. Therefore, measures must be taken to prevent humans and animals from being infected by these parasites.
